# Segment-based myocardial T1 and T2 mapping at 3T: feasibility and normal values

**DOI:** 10.1186/1532-429X-15-S1-P37

**Published:** 2013-01-30

**Authors:** Florian von Knobelsdorff-Brenkenhoff, Matthias A Dieringer, Marcel Prothmann, Andreas Greiser, Thoralf Niendorf, Jeanette Schulz-Menger

**Affiliations:** 1 Berlin Ultrahigh Field Facility, Berlin, Germany; 2 Working Group Cardiovascular MRI, Experimental and Clinical Research Center (Charite, MDC) and HELIOS Clinics, Berlin, Germany; 3 Siemens Healthcare, Erlangen, Germany; 4 Experimental and Clinical Research Center (Charite, MDC), Berlin, Germany

## Background

Myocardial T1 and T2 mapping using cardiovascular magnetic resonance imaging (CMR) is promising to improve disease detection and monitoring. We applied T1 and T2 mapping at 3T to study the technical feasibility and provide reference values in healthy volunteers.

## Methods

Sixty healthy volunteers (30 males / 30 females, 20 in each age group: 20-39 years, 40-59 years, 60-80 years) underwent T1 and T2 mapping of the left ventricle in 3 short axis slices. For T2 maps, 3 single shot steady state free precession (SSFP) images with different T2 preparation times (0, 24, 55ms) were acquired (TE 1.0 ms, TR 2.4 ms, voxel 1.9 x 1.9x6 mm^3^). For T1 maps, Modified Look-Locker Inversion Recovery (MOLLI) technique with 11 single shot SSFP images was used before and after injection of gadolinium contrast (pre-contrast: TE 1.0 ms, TR 2.6 ms, voxel size 1.4-1.7 x 1.4-1.7 x 6 mm^3^). T1 and T2 relaxation times were quantified for each slice and each myocardial segment.

## Results

**Figure 1:**
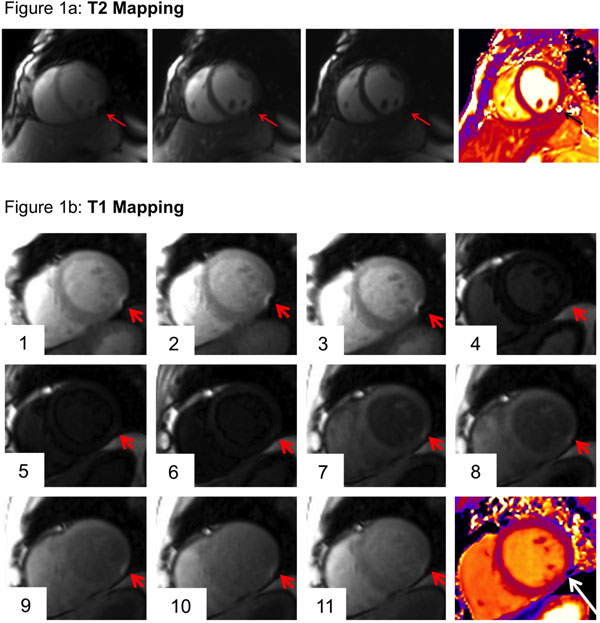
A T2 mapping; three original images and the resulting T2 map with susceptibility artifact in the inferolateral segment. b: T1 mapping; eleven original images and the resulting T1 map with susceptibility artifact in the inferolateral segment.

**Figure 2:**
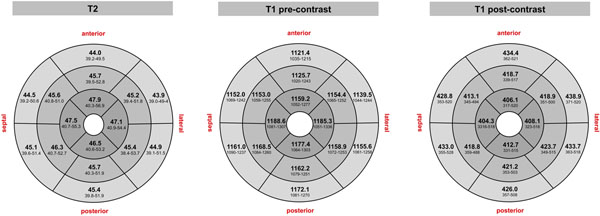
T2 and T1 relaxation times for each myocardial segment (mean, 95% tolerance interval).

With T2 maps, 97.7% of all segments were diagnostic and 2.3% were excluded (susceptibility artifact, Figure 1a). With T1 maps (pre-/post-contrast), 91.6% / 93.9% were diagnostic, while 8.4% / 6.1% were excluded (7.7% / 3.2% susceptibility artifact (Figure 1b); 0.7% / 2.2% incorrect motion correction; 0% / 0.7% mistriggering). Mean T2 times and 95% tolerance interval were: base: 44.1ms (39.3-49-5 ms); middle: 45.1 ms (39.9-50.1 ms); apex: 46.9 ms (40.8-53.8 ms). Mean T1 times and 95% tolerance interval pre- and post-contrast were: Base: 1157.1 ms (1074.5-1246.0) and 427.3 ms (363.2-502.7 ms). Middle: 1158.7 ms (1074.0-1250.1 ms) and 411.2 ms (349.9-483.2 ms). Apex: 1180.6 ms (1073.9-1297.9 ms) and 399.7 ms (323.0-494.6 ms). The segmental results are depicted in figure 2. Inter- and intra-observer analysis of T2 (r=0.95; r=0.95) and T1 (r=0.91; r=0.93) demonstrated excellent agreement (p<0.0001).

## Conclusions

T2 and T1 mapping at 3T was technically feasible, reference values for each myocardial segment are now provided, and observer dependency was low. However, 3T-related susceptibility artifacts and the relatively wide tolerance interval of T2 and T1 times must be considered during interpretation.

## Funding

This project is supported by the Else Kröner-Fresenius Stiftung (Bad Homburg, Germany).

